# Medical Unity in New York

**Published:** 1905-12

**Authors:** 


**A Monthly Review of Medicine and Surgery.**

*EDITOR:*

WILLIAM WARREN POTTER, M. D.

All communications, whether of a literary or business nature, books for review and exchanges, should be addressed to the editor 284 FRANKLIN ST., BUFFALO, N. Y.

VOL. LXI. DECEMBER, 1905. NO. 5

### Medical Unity in New York.

AFTER twenty-two years of separation, not to say dissention, the Medical Society of the State of New York and the New York State Medical Association at last have agreed to unite on what appears to be a permanent basis. It has taken four years of hard work by the joint committee of conference to effect the merger, but it is well worth the expenditure of the time, money, and patience which have necessarily been made to establish medical union in the Empire State.

Four years ago, Dr. H. L. Elsner of Syracuse, then president of the society, at the annual dinner of the ex-presidents, introduced a resolution, which at first met scant favor, authorising the president to make overtures to the association with a view to amalgamation. After considerable debate, however, the proposition carried, and the next day it became a part of the president's inaugural address. The society, by unanimous vote, indorsed the scheme, whereupon, the following named committee of conference was appointed: Henry L. Elsner, Syracuse, chairman; A. Jacobi, and A. M. Phelps, New York; A. Vander Veer, Albany, and George Ryerson Fowler, Brooklin. The death of Dr. Phelps soon afterward led to the appointment of Frank Van Fleet to fill the vacancy.

The Association met the action of the Society by the appointment of a committee of five—namely, E. Eliot Harris, chairman, William H. Biggam, Emil Mayer, Parker Syms, and Frederick Holme Wiggin, all of New York. The Society committee remains unchanged up to the present time, but the Association committee is constituted at present as follows: E. Eliot Harris, chairman, Julius C. Bierworth, Alexander Lambert, Parker Syms, and Wisner R. Townsend, all of New York.

These committees acting together as a joint committee of conference have worked out all the problems relating to the business affairs of the two organisations, which were many and often of a perplexing character; but through energy and perseverance, by day and by night, all difficulties have been surmounted and an understanding finally has been reached. The legal aspects of the subject, too, have been solved, and Judge Davy, at a special term of the supreme court held at Rochester, December 9, 1905, signed the decree consolidating the Medical Society of the State of New York and the New York State Medical Association, thus ending the unfortunate medical schism which has existed in this state for nearly a quarter of a century.

The Medical Society of the State of New York, as thus consolidated, will celebrate its centenary, January 30-31, and February 1, 1906, and the occasion may well be made one for rejoicing by a reunited and rehabilitated profession. It is an event to look forward to with absorbing interest and should mark an epoch in the medical affairs of this great commonwealth. Let us be thankful to each and every member of the joint committee that has wrought this Herculean task, and to everyone who has contributed to its happy culmination by aid, comfort, and advice.

The following account of the first meeting authorised by law under the amalgamation will prove of interest. It is taken from the *Journal of the American Medical Association*, December 23, 1905.

The new house of delegates of the Medical Society of the State of New York met in Albany, December 14, including the chairmen of the standing committees and the joint committee of ten. The secretary was ordered to notify all county societies of the fact that they should reorganise so as to agree with the constitution and by-laws, which was part of the agreement of amalgamation. All association members were also to be notified. The counsel who made application for consolidation gave the opinion that the order from the Supreme Court judge made all members of the New York State Medical Association who are in good standing members of the Medical Society of the State of New York on the receipt of a certificate of the president and secretary of the state association. It was decided to continue the journal and the medical directory and to continue the malpractice defense for the members of the Medical Society of the State of New York. The president and secretary were ordered to continue the journal for January and February, and a committee to be appointed by the president is to select an editor and to report at the annual meeting of the House of Delegates next month. A committee of five was appointed, of which the president is one, to carry out the referendum as per Section 7 of the court order relating to the Principles and Ethics of the American Medical Association. The constitution of the American Medical Association provides that when any state medical society is reorganised according to the plan of the American Medical Association, and by resolution expresses its allegiance to that body, it then becomes a constituent branch of it. Such a resolution was unanimously carried. The per capita assessment on members of the societies was placed at $3.00. A tentative constitution and by-laws for county societies was submitted as being suggestive and which might be adopted with very slight alteration by the several county medical societies of the state.

But we must not forget, in the haste of the moment, to accord credit to two men above all others, for laying the foundation of medical unity in the State of New York. We refer to Dr. Lewis S. McMurtry, of Louisville, reigning president of the American Medical Association, and Dr. Charles A. L. Reed, of Cincinnati, a former president of that body. Both of these gentlemen attended a meeting of the Society fifteen years ago, read papers and took active part in the proceedings. They made the acquaintance of the Society, its members, and methods, and have taken an active interest in its affairs since that time. They have visited it occasionally since and through these visits resolved to do all in their power to end the dual systems of state medical organisations.

Dr. Reed has been an aggressive opponent of the "old code" and never ceased his warfare until it was removed from the record books. Dr. McMurtry has been all the years mentioned a coadjutor and adviser of Dr. Reed, and has lent both activity and sympathy to the principles that have finally prevailed to restore a unity of medical government in New York. All honor, then, and all praise to these two distinguished members of the American medical profession, both of whom will visit Albany next month and participate in our centenary meeting.

---

A successful medical practitioner of many years standing makes the following statement: "There are a large majority of combinations which extemporaneous pharmacies cannot prepare properly; and I know that through the dishonesty, ignorance, or indifference of many retail druggists we are not able to get on prescriptions the very best drugs; hence it is to the manufacturing pharmacist, whose best interest lies in the purity and uniformity of his product, that we must look for our most reliable remedies.

"I endorse worthy proprietaries, but I most heartily condemn the great tendency of the "half-baked," so called manufacturing "chemist," to foist upon the profession and public cheap imitations of standard preparations."

The sudden and tragic death of Mr. James Russell Parsons in Mexico is an incident commanding somewhat more than passing regret. Mr. Parsons was a man of high attainments and ability, who for a number of years rendered important and lasting services to the cause of education in this state, who had more recently served the nation as consul general with fidelity and discretion, and who personally posessed many engaging traits of character and lived a blameless life. His death is a private bereavement and a public loss.—*New York Tribune.*

